# Cytology-based Cancer Surgery of the Head and Neck (CyCaS-HN): a prospective, randomized, controlled clinical trial

**DOI:** 10.1007/s00405-022-07333-7

**Published:** 2022-03-19

**Authors:** Maximilian Linxweiler, Sandrina Körner, Silke Wemmert, Hugo Rimbach, Johanna Helfrich, Barbara Linxweiler, Zoltan Ferenc Takacs, Erich Franz Solomayer, Mathias Wagner, Luc G. T. Morris, Bernhard Schick, Jan Philipp Kühn

**Affiliations:** 1 Department of Otorhinolaryngology, Head and Neck Surgery, Saarland University Medical Center, Kirrbergerstr. 100, building 6, 66421 Homburg, Germany; 2 Department of Gynecology, Obstetrics and Reproductive Medicine, Saarland University Medical Center, Homburg, Germany; 3 Department of General and Surgical Pathology, Saarland University Medical Center, Homburg, Germany; 4 Department of Surgery, Memorial Sloan Kettering Cancer Center, New York City, NY USA; 5 Immunogenomics and Precision Oncology Platform, Memorial Sloan Kettering Cancer Center, New York City, NY USA

**Keywords:** Head and neck cancer, Oral cancer, Liquid-based cytology, Tumor resection, Clinical trial

## Abstract

**Purpose:**

Liquid-based cytology (LBC) is routinely used in gynecology but is rarely applied in head and neck oncology though many suspicious lesions are easily accessible. While several studies have evaluated the potential use of LBC for early detection and molecular characterization of head and neck squamous cell carcinomas (HNSCCs), no study investigated its potential role in surgical management and therapy planning so far.

**Methods:**

Twenty-five patients with cT1-2 squamous cell carcinomas of the oral cavity and oropharynx were prospectively enrolled in this study and were randomized to two treatment arms: in the control arm, a diagnostic panendoscopy with incisional biopsy was followed by a second operation with transoral tumor resection ± neck dissection and tracheostomy. In the intervention arm, patients underwent LBC diagnostics and in case of a positive result received one single operation with panendoscopy and incisional biopsy for confirmation of LBC result by rapid section histology followed by transoral tumor resection ± neck dissection and tracheostomy in the same session.

**Results:**

Time between clinical diagnosis and definitive surgical treatment was significantly shorter in the intervention group compared with the control group (*p* < 0.0001). Additionally, time of hospitalization (*p* < 0.0001) and cumulative operation time (*p* = 0.062) were shorter in the intervention group. No significant differences in overall, progression-free, and disease-specific survival were observed.

**Conclusion:**

Cytology-based cancer surgery is a promising therapeutic strategy that can potentially be considered for a well-defined group of early-stage HNSCC patients and help to avoid repetitive general anesthesia, shorten the diagnosis-to-treatment interval and spare operation as well as hospitalization time.

## Introduction

Head and neck squamous cell carcinomas (HNSCC) represent the eighth most common cancer worldwide and accounted for more than 444,000 deaths in 2020 [1]. Despite major therapeutic advances in advanced staged disease, including the approval of pembrolizumab [2, 3] and nivolumab [4] for recurrent and/or metastatic HNSCCs in the first and second line, the prognosis of patients with HNSCC has not markedly changed over the past decades. Currently, 5-year overall survival rates remain unchanged at 55–65% [5]. One major reason for this poor prognosis is delayed diagnosis, since most patients are diagnosed with tumors having reached a locoregionally advanced stage, often with lymph node metastases [6]. These factors are both prognostically negative and also limit therapeutic options. Additionally, HNSCC patients often have a history of extensive tobacco and/or alcohol consumption and suffer from comorbidities including chronic obstructive pulmonary disease, chronic heart failure, coronary artery disease and hypertension [7].

Considering these diagnostic and therapeutic challenges, we have developed an approach of liquid-based cytology (LBC) for the non-invasive early detection of HNSCC. Here, we present the results of our clinical trial of this approach, to determine if it is able to facilitate more effective therapeutic strategies with lower morbidity.

LBC was successfully introduced in gynecology more than 20 years ago and has proven its enormous value for the prevention and early detection of dysplastic and cancerous lesions of the uterine cervix as well as after care of cervical cancer patients [8, 9]. Compared to conventional swab cytology, LBC bears several advantages, including markedly better quality of cell preparation, optimized visualization of cell morphology and a clean background. In gynecology, it is considered the gold standard for cytological diagnostics [10, 11]. Though the majority of HNSCCs are comparably easily accessible for this technique, LBC is rarely used for the diagnostic work-up of suspicious lesions of the oral and/or pharyngeal mucosa. LBC is not mentioned in the European or US guidelines for diagnosis and treatment of head and neck cancers [12, 13]. Several groups have investigated the diagnostic value of LBC in head and neck oncology and reported a sensitivity of 75–97.5% and a specificity of 50–99% for diagnosing high-grade dysplasia and squamous cell carcinomas of the oral cavity and oropharynx [14–20]. Compared with conventional swab cytology LBC has demonstrated better diagnostic performance and also enables further molecular diagnostics e.g. HPV-DNA-PCR and p16-Ki67, dual immunocytochemical staining for assessing HPV status as an important prognostic and predictive biomarker in oropharyngeal cancer patients [21]. To date, no clinical study has investigated a potential use of LBC for guiding surgical treatment of early-stage HNSCC patients, as compared against the established standard of diagnostic panendoscopy with incisional biopsy of the primary tumor, followed by tumor resection in a second step under general anesthesia [12, 13].

Against this background, we initiated the CyCaS-HN study as a monocentric, prospective, randomized controlled clinical trial to evaluate the new concept of **Cy**tology-based **Ca**ncer **S**urgery of the **H**ead and **N**eck. The CyCaS-HN study aimed to investigate a potential use of LBC in early-stage HNSCC patients for shortening the time between clinical diagnosis and definitive surgical treatment and combining diagnostic panendoscopy and tumor resection in one surgical procedure to spare the patients repetitive hospital stays and general anesthesia.

## Materials and methods

### Patients and clinical data

Head and neck squamous cell carcinoma patients were prospectively recruited for the CyCaS-HN study at the Department of Otorhinolaryngology, Head and Neck Surgery of a University Medical Center between July 2017 and May 2021. Inclusion criteria were the clinical diagnosis of a cT1-2 cN0-3 cM0 squamous cell carcinoma of the oropharynx and/or the oral cavity, adequate accessibility of the primary tumor for a brush biopsy and age ≥ 18 years. Exclusion criteria were cases requiring reconstruction with a locoregional and/or distant flap, a tumor location outside the oral cavity or oropharynx, limited accessibility of the primary tumor for a brush biopsy, non-surgical treatment plan, pregnancy, and age < 18 years. The time between clinical diagnosis and definitive surgical treatment was defined as primary target criterion. Secondary target criteria included time of hospitalization, progression-free survival (PFS), disease-specific survival (DSS), and overall survival (OS). In total, 30 patients meeting the inclusion criteria were screened for participation in this prospective clinical trial and 25 of these were finally included. Five patients refused participation after careful information about the study design and randomization. The final study population comprised 17 males and 8 females with a mean age of 63.76 years. Clinical data of all patients including tumor location, T and N stages, p16 status and choice of adjuvant treatment are shown in Table 1.

**Table 1:** Clinical data of included patients

	Intervention group	Control group	Total
No. of patients	11	14	25
Sex			
Male	10 (91%)	7 (50%)	17 (68%)
Female	1 (9%)	7 (50%)	8 (32%)
Mean age	64.64	63.07	63.76
Localization			
Tonsil	3 (36%)	6 (43%)	9 (36%)
Tongue base	0	3 (21%)	3 (12%)
Tongue	7 (64%)	4 (29%)	11 (44%)
Soft palate	1 (9%)	1 (7%)	2 (8%)
T stages			
T1	7 (64%)	6 (43%)	13 (52%)
T2	4 (36%)	8 (57%)	12 (48%)
N stages			
N0	6 (55%)	8 (57%)	14 (56%)
N1	2 (18%)	2 (14.5%)	4 (16%)
N2a	1 (9%)	1 (7%)	2 (8%)
N2b	2 (18%)	2 (14.5%)	4 (16%)
N3	0	1 (7%)	1 (4%)
p16 status			
Positive	5 (45%)	6 (43%)	11 (44%)
Negative	6 (55%)	8 (57%)	14 (56%)
Adjuvant treatment			
None	5 (45%)	5 (36%)	10 (40%)
RT	2 (18%)	3 (21%)	5 (20%)
CRT	4 (37%)	6 (43%)	10 (40%)

This prospective clinical study was in accordance with the ethical standards of the institutional and national research committees and with the 1964 Helsinki Declaration and its later amendments or comparable ethical standards. All patients gave their informed consent for the use and publication of their clinical data and tissue samples. The CyCaS-HN study has been approved by the local Medicines’ Association ethics review committee (index number 68/17) and was registered at the German Clinical Trials Register (DRKS-Nr. 00,013,507) prior to inclusion of the first patient.

### Study arms and randomization

**Fig. 1:**
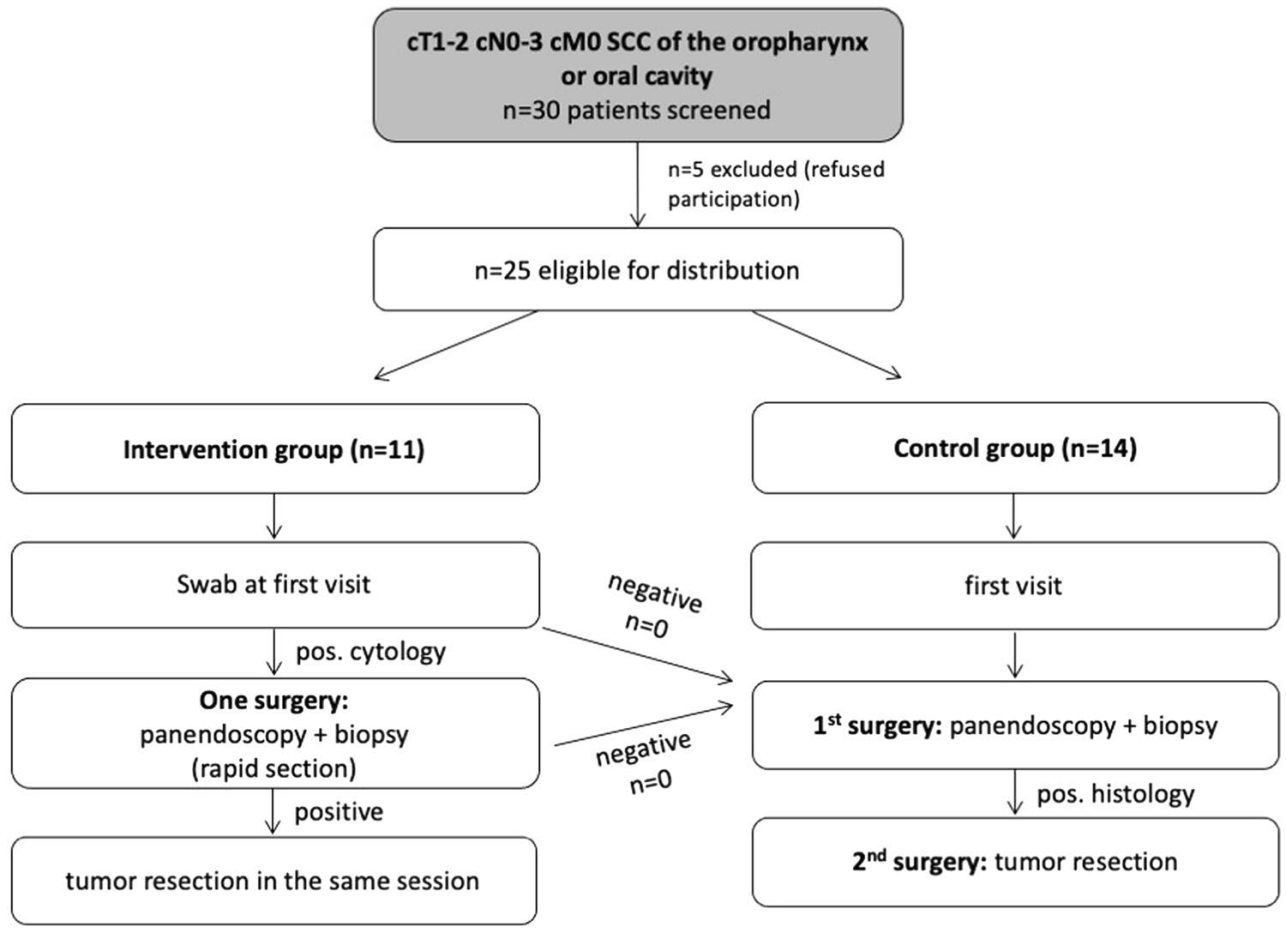
Study flowchart. Thirty patients meeting the inclusion criteria were screened for inclusion into the study and finally 25 patients were distributed to the intervention group (*n* = 11) or the control group (*n* = 14)

Distribution of the included patients to the two study arms (intervention group, control group) was performed using the stratified randomization technique considering age, p16 status, T and N stage as well as the choice of adjuvant treatment as covariates. This randomization technique is recommended especially for small study populations with less than 50 participants to achieve an optimal balancing of all covariates that are relevant for the main target criteria. The study was designed as non-blinded study. Of 30 screened patients, 5 did not consent to participate so that 25 patients meeting the inclusion criteria were eligible for randomization. Eleven patients were assigned to the intervention group and 14 patients were assigned to the control group. In the control group, patients were treated with a standard of care protocol starting with a diagnostic panendoscopy including an incisional biopsy for histopathological verification of diagnosis. In case of a result confirming carcinoma, tumor resection ± neck dissection and tracheostomy were performed. Both operations were performed under general anesthesia. In the intervention group, a brush biopsy for LBC diagnostics was performed at the patient’s first outpatient clinical presentation. In case of a positive result, one single surgical procedure was planned including a diagnostic panendoscopy with an incisional biopsy for rapid section histology followed by tumor resection ± neck dissection and tracheostomy in case of a confirmed positive result. In case of a positive LBC but negative rapid section histology, the operation was stopped after another 1–3 biopsies for FFPE (formalin-fixed paraffin embedded) histology awaiting the final histological report. In case of FFPE samples being negative as well, an individual plan depending on the local findings, imaging results and the patient’s symptoms was necessary e.g. including a PET-CT scan or another incisional biopsy. In both study arms, adjuvant treatment was planned according to the currently available guidelines after case discussion in an interdisciplinary tumor board. For all patients, a CT (computed tomography) scan of the neck, thorax and abdomen was performed as part of the diagnostic work-up prior to tumor resection. A study flow chart is shown in Fig. 1.

### Liquid-based swab cytology

Cytological brush biopsies were taken using the Medscand Cytobrush (Cooper Surgical Inc., CT, USA) at the first clinical presentation of the patients in the intervention group in an ambulatory setting. After gently wiping off the macroscopically suspect mucosal areas (the center of lesions in the case of solid tumors and the edges in the case of ulcerous tumors; *n* = 11 HNSCC patients), brushes were immersed and rinsed in a PreservCyt Solution vial (Hologic Inc., MA, USA). Afterwards, the cell suspensions were prepared on microscope glass slides using the ThinPrep®-system (Hologic Inc., MA, USA) according to the manufacturer’s instructions.

### Papanicolaou staining and cytopathological analysis

For morphological analysis, one cytological preparation for each case was Papanicolaou-stained using a standard protocol. As there are no worldwide accepted standard criteria for cytologic diagnosis of HNSCC, we applied the cytomorphological criteria for uterine cervical smears due to a very similar cytomorphological appearance. The slides were classified independently according to the Bethesda system by two technical assistants with wide experience in valuing pap smears of the uterine cervix as well as one cytopathologist. Only samples with at least one tumor cell (Bethesda system: HSIL (high-grade squamous intraepithelial lesion) or SCC (squamous cell carcinoma)) were assigned as positive result.

### Statistical analysis

GraphPad Prism 9.0 (GraphPad Software, La Jolla, CA, USA) and SPSS version 27 (IBM, Ehningen, Germany) were used for statistical analysis presuming a significance level of 5% (*α* = 0.05) and a statistical power of 80% (*β* = 0.8). The existence of normal distribution for the analyzed parameters was controlled by Kolmogorov–Smirnov test, Anderson–Darling test, D’Agostino–Pearson test, and Shapiro–Wilk test. Homogenous variance was checked by Levine test. If parameters showed no normal distribution in at least one of the aforementioned tests, non-parametric Mann–Whitney *U* test was used. In case of normal distribution in all of the aforementioned tests, a two-sided *t* test was used. For survival analyses, the Kaplan–Meier algorithm and the log-rank test were used. Correlation analyses between two dichotomous variables were performed using a Fisher’s exact test. *p* values are indicated in the figures.

## Results

### Correlation between cytology, rapid section histology and FFPE-based histology

**Fig. 2:**
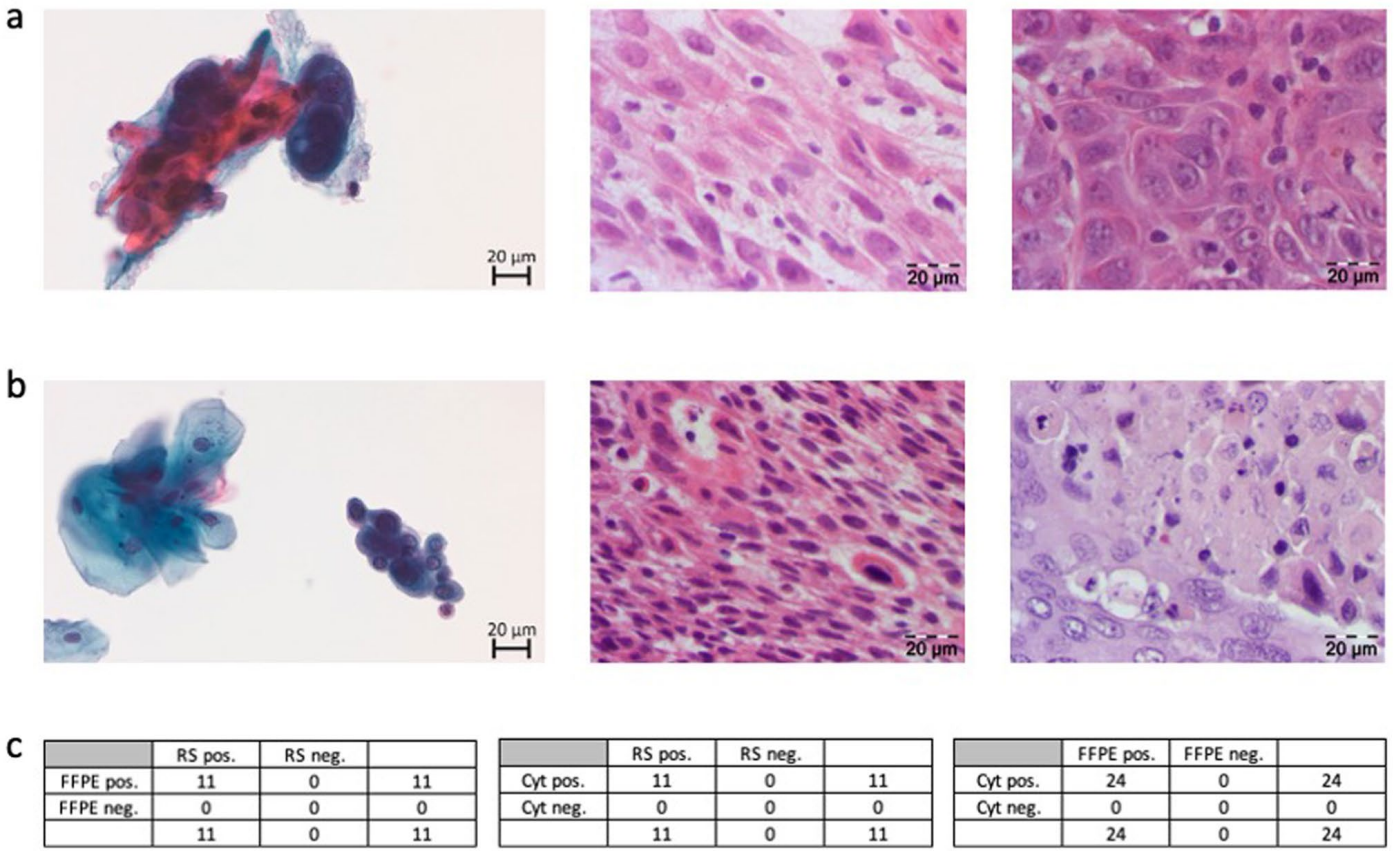
Representative cytological and histological images and correlation between cytology, rapid section histology and FFPE-based histology. **a** PAP-stained cytological image (left), H&E-stained rapid section histological image (middle), and H&E- stained FFPE histological image (right) of one representative patient with an HPV-negative tongue cancer. **b** PAP-stained cytological image (left), H&E-stained rapid section histological image (middle), and H&E- stained FFPE histological image (right) of one representative patient with an HPV-positive tonsil cancer. **c** Correlation between FFPE histology and rapid section histology (left), cytology and rapid section histology (middle), cytology and FFPE histology (right). In **c**, the number of patients with cancer diagnosis (positive) or without cancer diagnosis (negative) is indicated. *RS* raid section histology, *Cyt* cytology, *FFPE*, formalin-fixed paraffin embedded tissue histology

To evaluate the diagnostic sensitivity and positive predictive value of LBC for the patients included in this study, we compared the results of LBC with rapid section histology and FFPE histology as current diagnostic gold standard. Representative images of all three techniques are shown for a p16-positive and a p16-negative patient in Fig. 2a, b.

For the patients of the intervention group for whom LBC, rapid section histology and FFPE histology were available in every case, we found a concordance between LBC and rapid section histology as well as FFPE histology in all cases: tumor cells were found in all cytological and histological preparations (Fig. 2c). However, in one case, a first intraoperative rapid section histology was negative without detection of tumor tissue. Due to the positive LBC, we took another biopsy of the suspicious area and sent it out for rapid section histology that then showed a positive result. When comparing the results of LBC and FFPE that were available for all included patients, we again saw a congruence for all cases (Fig. 2c). Hence, LBC showed a sensitivity for detecting squamous cell carcinomas of 100% (24/24) with a positive predictive value of 100% (24/24) in this study.

### Time between diagnosis and treatment, cumulative operation and hospitalization time

**Fig. 3:**
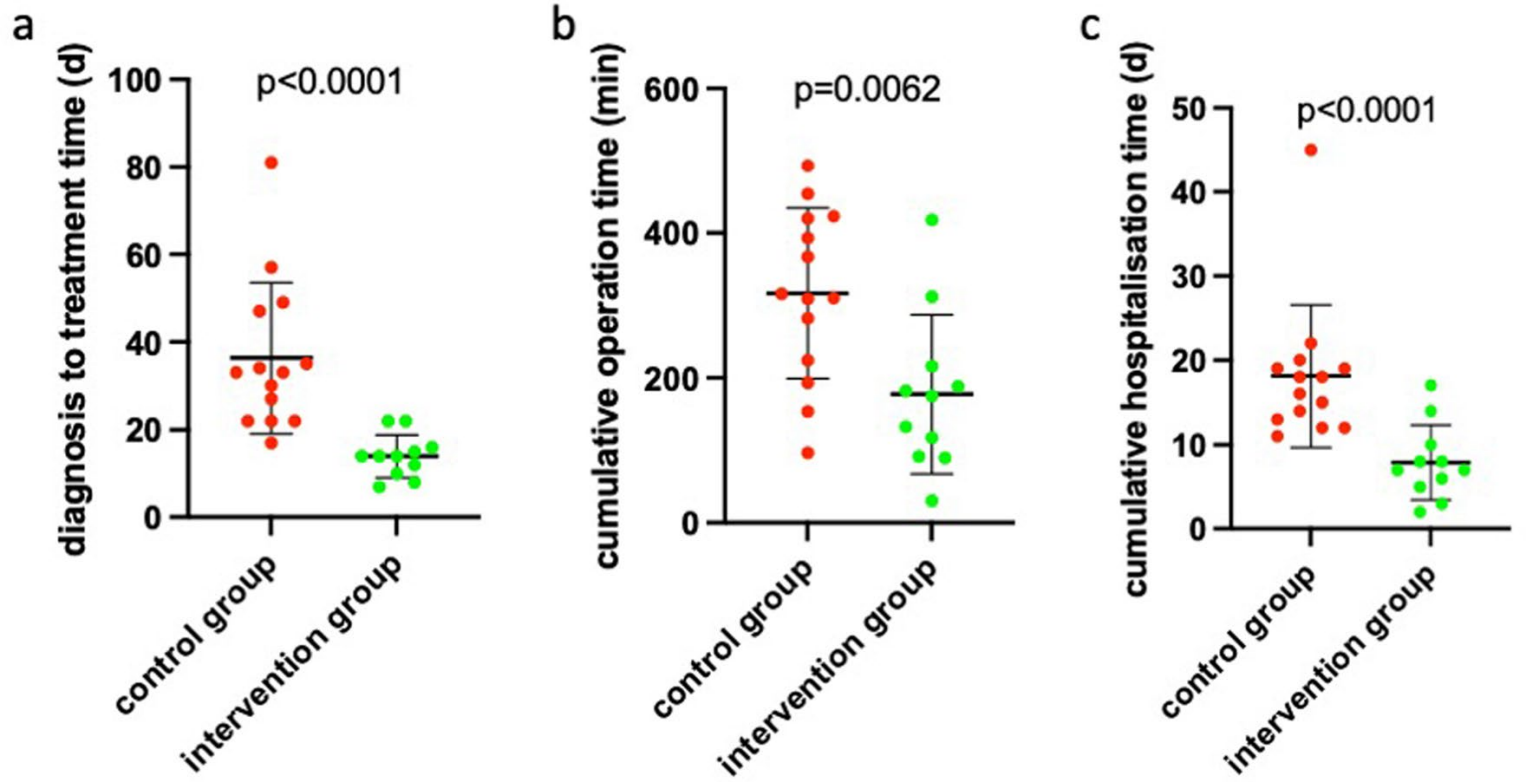
Time from clinical diagnosis to definitive cancer surgery in the control and intervention group, cumulative operation time and time of hospitalization. **a** Time between first clinical diagnosis and definitive surgical cancer treatment is indicated for the control group (red) and the intervention group (green). **b** Cumulative operation time is indicated for the control group (red) and the intervention group (green). **c** Cumulative hospitalization time is indicated for the control group (red) and the intervention group (green). In **a**–**c** every dot represents one patient, the arithmetic mean is indicated by a line. The error bars represent the standard deviation

As the primary outcome of our study, we defined the time between clinical diagnosis at the first visit in our clinic and the curative surgical procedure. When comparing this time span between both study groups we found a highly significant reduction in diagnosis-to-treatment time in the intervention group compared with the control group (*p* < 0.0001; Fig. 3a) with a mean time span of 14 days for the intervention group and 36 days for the control group and with a range between 7 and 81 days. As a secondary outcome, the cumulative operation time was significantly shorter in the intervention group (time of one single operation including panendoscopy, biopsy for rapid section histology, and tumor resection ± neck dissection and tracheostomy) compared with the control group (time of the panendoscopy with biopsy for FFPE histology + time of tumor resection ± neck dissection and tracheostomy; *p* = 0.0062, Fig. 3b). The mean operation time in the intervention group was 175 and 313 min in the control group with a range between 31 min for the resection of a cT1 cN0 cM0 tongue carcinoma and 493 min for resection of a cT2 cN2b cM0 tonsil carcinoma with bilateral neck dissection in levels II to V as well as tracheostomy, both with a preceding panendoscopy in the same (intervention group) or a second operation (control group). Additionally, cumulative hospitalization time was analyzed as a secondary target criterion and proved to be significantly shorter in the intervention group (mean: 7 days) compared to the control group (mean: 17 days; *p* < 0.0001, Fig. 3c) with a range between 2 and 45 days.

### Patient survival in the intervention and control group

**Fig. 4:**
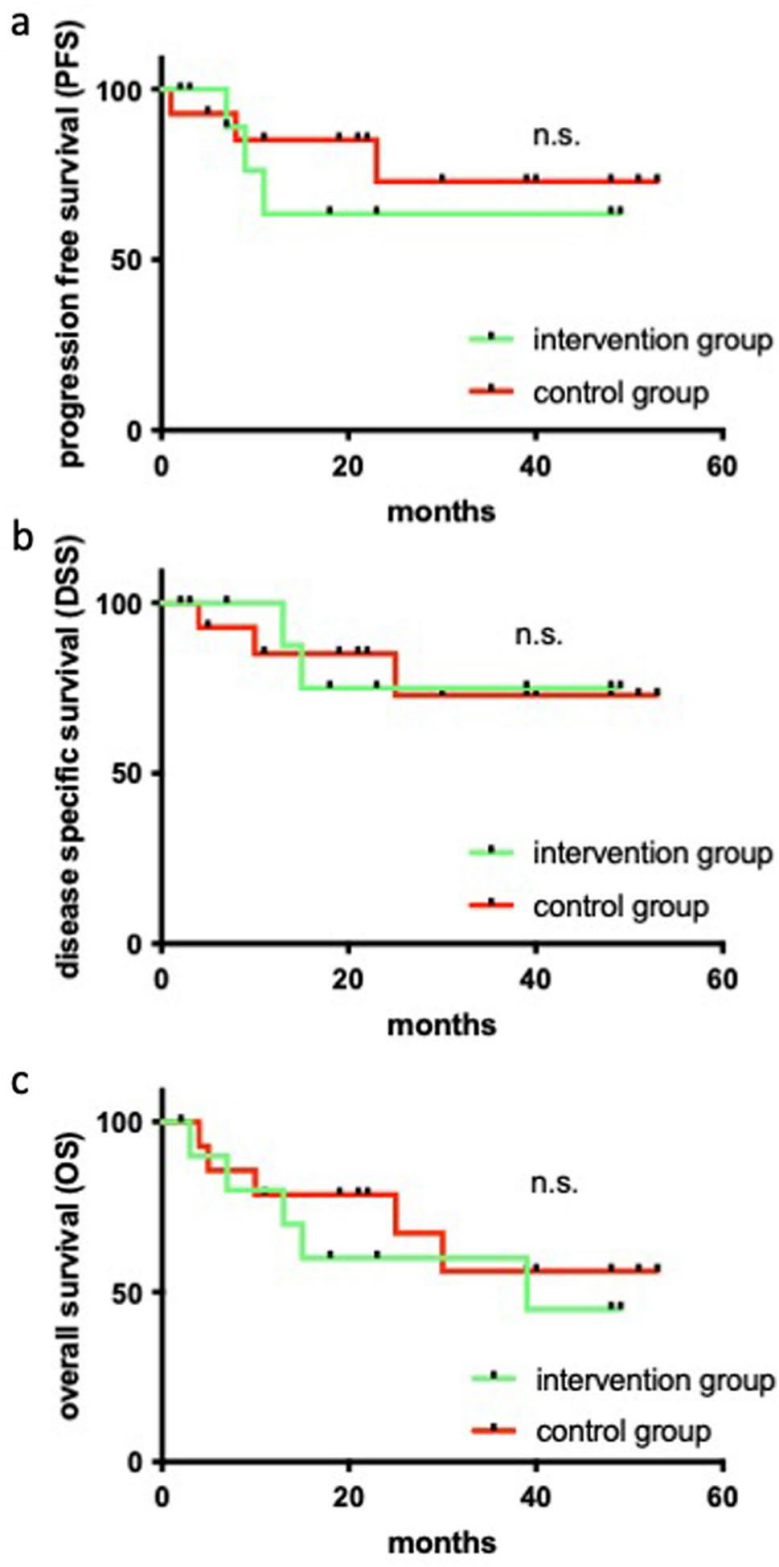
Progression free, disease-specific and overall survival for the patients of the control and intervention group. **a** Progression free survival (PFS) for the control group (*red*) and the intervention group (*green*). **b** Disease specific survival (DSS) for the control group (*red*) and the intervention group (*green*). **c** Overall survival (OS) for the control group (OS) and the intervention group (*green*). In **a**–**c** censored data are indicated by *black dots* on the survival curves; significance is indicated for each analysis (log-rank test); *n.s.* not significant

Next, we analyzed the progression-free survival (PFS), disease-specific survival (DSS) and overall survival (OS) for all included patients (Fig. 4). The median follow-up was 25.8 months ranging from 2 to 53 months. In the course of the study, five patients of the intervention group and five patients of the control group died (10/25, 40% of all included patients). Causes of death were pancreatic cancer with liver metastases, liver cirrhosis, septic shock due to staphylococcal infection, and recurrent tongue cancer with lung metastases (two cases) in the intervention group. In the control group, causes of death comprised acute myocardial infarction, severe stroke, recurrent tongue cancer with peritoneal carcinomatosis, recurrent cancer of the floor of the mouth and recurrent cancer of the tongue base. Locoregional tumor recurrence was observed in three patients in the control group and in none of the patients in the intervention group. Two patients of the intervention group and one patient in the control group developed distant metastases with localizations in the lung (*n* = 2, intervention group) and the peritoneal cavity (*n* = 1, control group).

Neither the progression-free survival (PFS) nor the disease-specific survival (DSS) showed a significant difference between the patients of the intervention and control group (Fig. 4a, b). Comparably, the overall survival (OS) was comparable between the intervention and the control group (Fig. 4c) with a 3-year overall survival rate of 63% (intervention group) and 64% (control group), respectively.

## Discussion

In the CyCaS-HN study, we investigated the new concept of **Cy**tology-based **Ca**ncer **S**urgery of the **H**ead and **N**eck in a prospective, randomized, controlled clinical trial design. With this new therapeutic concept, we aimed to achieve a shortening of time between clinical diagnosis and definitive surgical treatment by combining diagnostic panendoscopy and tumor resection in one surgical procedure after an initial LBC-based proof of clinical diagnosis at first clinical presentation. In total, 25 patients were included in the study an assigned to the intervention group (*n* = 11) or the control group (*n* = 14) and were surgically treated according to the study protocol. Final analysis showed that not only the time between clinical diagnosis and definitive surgical treatment but also the cumulative operation time and time of hospitalization was significantly shorter in the intervention group compared with the control group without any difference in PFS, DSS, and OS.

In the context of LBC in head and neck oncology, several studies investigated the diagnostic accuracy and validity of this technique for diagnosing HNSCCs [14] and also compared it with conventional swab cytology [15, 17, 20]. In these studies that comprised markedly larger patient cohorts compared to our study, sensitivities between 75 and 97.5% and specificities between 50 and 99% were reported [14–20]. The diagnostic sensitivity, specificity, and positive predictive value we found in our study range among the highest values reported in the aforementioned studies. However, one has to consider the low number of patients included in our study so that the indicated diagnostic values have to be treated with caution and need a confirmation in extended patient cohorts. However, we found a comparably high sensitivity, specificity, and positive predictive value when using the same LBC methodology on 65 patients in an earlier study so that we would not expect relevant discrepancies in larger study cohorts [21]. Consistently, all the aforementioned studies reported a higher quality for cytological preparations using LBC technique compared to conventional swabs with the main advantages of a clearer background and an optimized visualization of the epithelial cells allowing a more accurate assessing of potentially dysplastic or malignant cells [10, 11]. Apart from morphological evaluation of the cellular composition in the cytological sample, LBC can be used for molecular diagnostics including immunocytochemistry and PCR [21] e. g. for determining the HPV status with highly relevant prognostic and predictive implications [22, 23]. Despite these advancements in cytological techniques for early diagnosis of HNSCC patients, no study has addressed a potential use of cytology for therapeutic management of HNSCC patients so far.

Should such an approach be used for oral cancer screening? In comparison to gynecologic oncology where LBC is routinely used for cervical cancer screening, a screening approach in head and neck oncology does not seem to be reasonable based on the current state of evidence. The Global Oral Cancer Forum reviewed recent studies on screening approaches for oral cancer including conventional oral examination, vital rinsing and exfoliative cytology amongst others and found that there is insufficient evidence to show that oral cancer screening can reduce mortality from oral cancer, and to date, no country has implemented a formal oral cancer screening program [24]. Major reasons for this lack of evidence and validity of reported screening approaches are that (i) only few evaluations of screening programs have been published so far including only one randomized controlled trial [25] and that (ii) there is a lack of understanding of malignant transformation of oral potentially malignant lesions and disease progression [26]. Additionally, patient with a high risk for developing HNSCC do usually not visit a dentist or ENT doctor on a sufficiently regular basis to make screening approaches feasible. Hence, an application of LBC for early diagnosis of HNSCC as proposed in our study represents a more promising approach. In this context, the Cochrane Collaboration reviewed non-invasive diagnostic tests for oral cancer and potentially malignant disorders in patients presenting with clinically evident lesions and reported a sensitivity of 90% and specificity of 94% for oral cytology ranking the highest among all investigated diagnostic methods [27]. Therefore, they stated a high potential for oral cytology as a promising method for early detection of oral cancer [27]. The advantages also mentioned in this review, i.e. being a less invasive procedure, providing immediate results, being less painful for the patient and requiring no general anesthesia were the major benefits we took advantage of in our study in the context of surgical treatment planning.

There are several caveats when considering our results. The number of included patients in our study is comparably low due to the inclusion criteria that do not include patients with several subtypes of HNSCC. The reason for these comparably strict inclusion criteria are that (i) in hypopharyngeal and laryngeal cancer patients the primary tumor region cannot be accessed with a cytological brush when the patient is awake so that cytological diagnostics are not feasible in an ambulatory setting and (ii) we could not exactly calculate the risk of false positive cytopathological results, which would imply the consequence that a large tumor resection would have turned into a diagnostic panendoscopy with incisional biopsy. Therefore, we excluded tumor resections with a need for plastic reconstruction using locoregional or free flaps to avoid an excessive loss of OR capacity. Though the results for predefined primary and secondary target criteria are highly significant an extension of the study group is highly desirable to increase the validity of our study also with regard to the validity of the indicated diagnostic accuracy of LBC. As a high number of screened patients will be necessary due to the stringent inclusion criteria more participating centers will have to collaborate to continue the study in a multicentric study design. Accordingly, an extension of the CaCaS-HN study to a multicentric study design due to the promising preliminary results of this first monocentric experience is planned to enlarge the number of included patients and hopefully confirm our reported results with a higher level of evidence. The presented first interim report of the CyCaS-HN study aims to draw attention to this study design and show that the interventional concept of cytology-based cancer surgery of the head and neck works in clinical practice with highly significant results though of the comparably low number of included patients. Another point is that the comparably wide spectrum of surgical treatment ranging from a simple wedge excision of a T1 tongue cancer up to a transoral resection of a T2 tongue base tumor with bilateral neck dissection and a tracheostomy represents a potential bias for the predefined primary and secondary target criteria. For that reason, we randomized patients to the intervention and control arm considering p16 status, choice of adjuvant therapy, T status and N status as covariates in order to achieve a best possible balancing in terms of molecular and clinical risk factors as well as metastastic load (see Table 1). However, there are several other factors that could potentially have influenced the predefined target criteria e.g. experience of the surgeon, surgical technique (cold steel vs. monopolar vs. CO_2_ laser resection) and intensity of intraoperative bleeding, wound healing, recovery of swallowing function, home management of tracheostomy and patient compliance. These factors all represent potential biases. To achieve a best possible balancing of these factors between both study groups, a larger number of patients is necessary which can ideally be realized for following studies in a multicentric study approach.

Considering the diagnostic accuracy of cytopathology, we acknowledge that a correct cytopathological diagnosis is highly dependent on the experience of the cytopathologist especially as oral and/or pharyngeal cytopathology is not frequently used in clinical practice. As our group works on LBC diagnostics in head and neck cancer patients for many years and routinely uses this technique in clinical practice, it may be the case that less experienced centers may encounter a learning curve with this technique. Therefore, we recommend the strategy of cytology-based head and neck cancer surgery when a trained cytopathologist is available and is introduced to this therapeutic concept. As Pandey et al. [28] found a high level of concordance without any significant difference when evaluating the performance of cytotechnologists and cytopathologists in assessing the adequacy and preliminary diagnostic accuracy of oral brush liquid-based cytology, it represents an alternative to involve experiences cytotechnologists in the evaluation of oral LBC specimens when a trained cytopathologist is not available. Even an automated analysis of LBC samples of the head and neck region using artificial intelligence has been investigated with promising preliminary results giving reasons for further studies [29]. In this context, it has to be mentioned that no standardized pathological reporting system with uniform assessing criteria exists for LBC of the oral and pharyngeal mucosa. However, first studies showed that the Bethesda system of reporting for cervical cytology that we also used in our study can easily be applied to LBC specimens of the oral mucosa [30] and also be modified to enhance diagnostic accuracy for the head and neck region [31] due to the high accordance in cytomorphological findings as compared with the uterine cervix. Nevertheless, a unique cytopathological reporting system for head and neck oncology should be pursued which can only be facilitated by generating a high amount of cytological and respective clinical follow-up data of large patient cohorts. Additionally, other histopathological findings apart from squamous cell carcinomas that represent far more than 90% of all head and neck malignancies including lymphoma, mucosal melanoma, and adenocarcinoma could pose challenges for a correct LBC-based diagnosis. Furthermore, one has to consider potential sampling errors in collecting cells of suspicious lesions with a brush when evaluating LBC findings. It cannot be excluded that malignant cells from adjacent areas of the oral resp. pharyngeal mucosa can float into the swabbed area and thereby generate false positive cytopathological results. However, we never observed a comparable case in our present and previous studies using LBC. Nonetheless, the aforementioned limitations of LBC illustrate why LBC will probably never be able to completely replace a histopathological examination as an essential backbone of surgical therapy. Therefore, we strongly recommend always to confirm a LBC-based HNSCC diagnosis by rapid section histology or FFPE-based histology as proposed in the intervention arm of our study.

Taken together, we found in our study that the concept of cytology-based surgery of head and neck cancer represents a promising therapeutic strategy that can potentially be considered for a well-defined group of early-stage HNSCC patients and help to avoid repetitive general anesthesia, shorten the diagnosis-to-treatment interval and spare operation as well as hospitalization time. An extension of the CyCaS-HN study to a multicentric design is planned for the near future in order to confirm the promising results of this first interim report in a monocentric setting with a higher level of evidence.
